# Supplementary material for: Golgi stress induces SIRT2 to counteract Shigella infection via defatty-acylation
Source: Nat Commun. 2022 Aug 2;13:4494. doi: 10.1038/s41467-022-32227-x (PMC9345896; doi:10.1038/s41467-022-32227-x)
Supplement: Supplementary file 1 — Supplementary Information [file 41467_2022_32227_MOESM1_ESM.pdf]

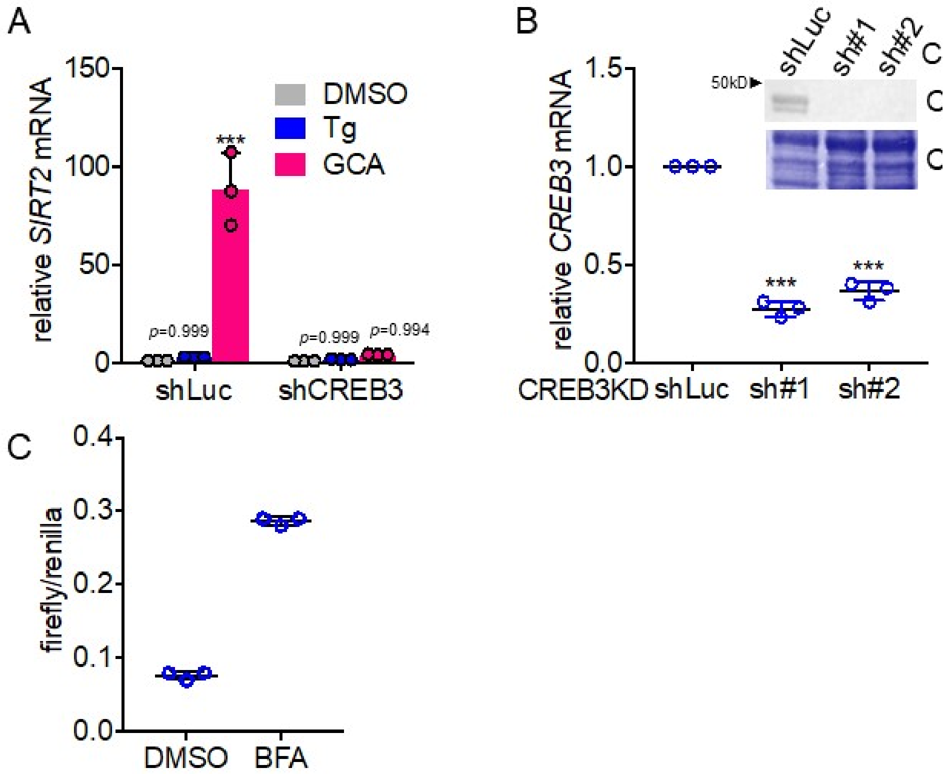

Supplementary Figure 1. CREB3 promotes SIRT2 transcription under Golgi stress.
(A) RT-PCR analysis of *SIRT2* mRNA levels in A549 *CREB3* KD cells treated with 1 μM Thapsigargin (Tg) or 5 μM Golgicide A (GCA). mRNA was normalized to DMSO-treated shLuc (control) cells. n=3 biological replicates. (B) *CREB3* knockdown is confirmed by RT-PCR and western blot. mRNA level was normalized to shLuc (control) cells. n=3 biological replicates. Statistical evaluation was done by two-way ANOVA. (C) SIRT2-promoter driven firefly transcription in cells under BFA treatment. The Renilla luciferase construct was used as an internal control. n=3 biological replicates. Data are represented as mean ± SEM. Statistical evaluation was done using unpaired two-tail Student's t test. ****p<0.001.*

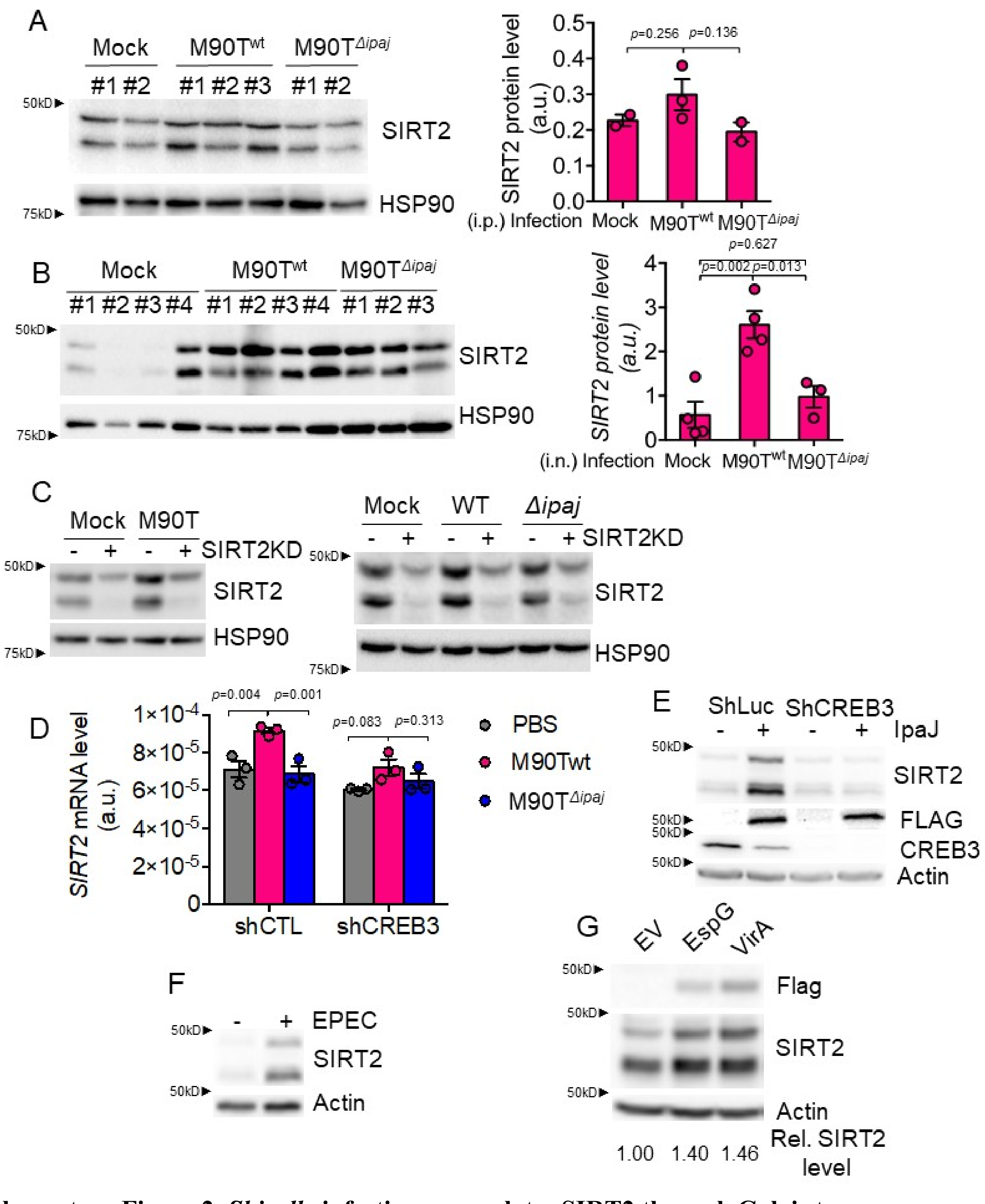

Supplementary Figure 2. ***Shigella*** **infection upregulates SIRT2 through Golgi stress.**
(A) Immunoblots of Sirt2 in colon tissue from 6-week-old wildtype C57BL/6J mice that were intraperitoneally infected with mock, wildtype or IpaJ deletion *S. flexneri* M90T strains. The quantification of SIRT2 protein levels, normalized to HSP90, is shown on the right. n=3 biological replicates. (B) Immunoblots of Sirt2 in bronchoalveolar lavage cells from 6 to 8-week old wildtype C57BL/6J mice intranasally infected with mock, wildtype or IpaJ deletion S. flexneri M90T. Quantification of SIRT2 protein levels (normalized to HSP90) in the immunoblots is shown on the right. n=3 biological replicates. Statistical evaluation was done by one-way ANOVA. (C) *Shigella* infection

upregulates SIRT2 in an IpaJ-dependent manner in A549 cells. Left: Immunoblots of SIRT2 in A549 cells infected with mock or *S. flexneri* M90T for 4 hrs. Right: Immunoblot of SIRT2 in A549 control and SIRT2 KD cells infected with mock or equal number of wildtype or IpaJ deletion *S. flexner*i M90T cells for 4 hrs. n=3 biological replicates. (D) RT-PCR analysis of *SIRT2* in A549 *CREB3* knockdown cells infected with mock, equal number of wildtype or IpaJ deletion *S. flexneri* M90T cells for 4 hrs. mRNA level in each sample was normalized to internal control (*Actb*). (E) Immunoblots of control and shCREB3 A549 cells transfected with IpaJ to assay SIRT2 protein level upregulation. (F) Immunoblots of A549 cells infected with enteropathogenic *E. coli* for 18 hours to assay SIRT2 protein level regulation. (G) Immunoblots of HEK 293T cells transfected with empty vector or flag tagged EspG or VirA. Statistical evaluation was done by two-way ANOVA. Data are represented as mean ± SEM.

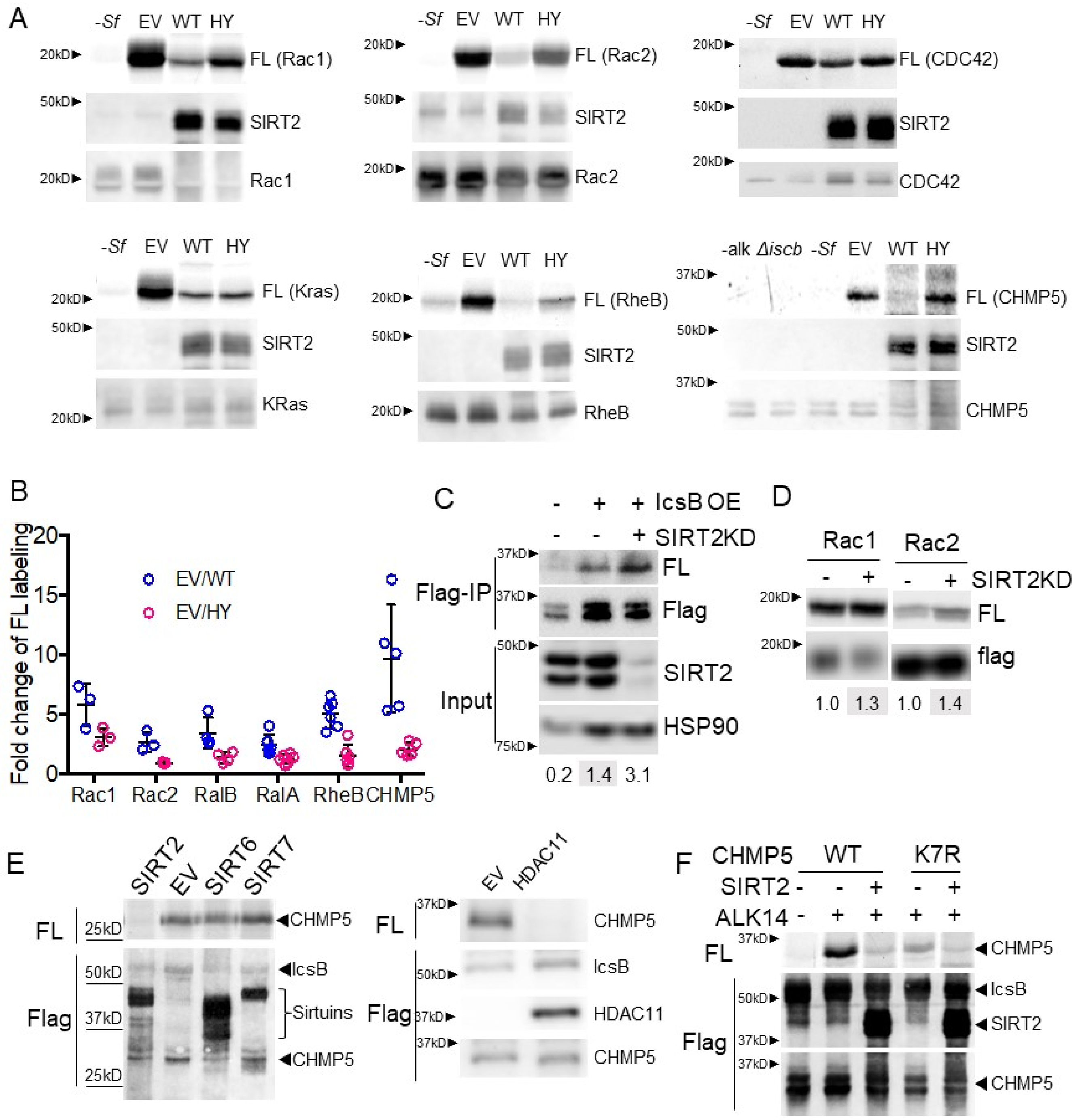

Supplementary Figure 3. SIRT2 is a potent lysine defatty-acylase that counteracts the action of IcsB.

(A) In-gel fluorescence detection of lysine fatty acylation of Flag-tagged IcsB substrates in HEK293T cells that are infected with *S. flexneri* M90T. -alk, sample without Alk14 treatment. Δicsb, sample with *S. flexneri* IcsB deletion strain infection. -*Sf*, sample without S. *flexneri* infection. EV, sample with empty vector expression. WT, sample with SIRT2 WT expression. HY, sample with SIRT2 HY mutant expression. FL, fluorescence (indicative of lysine fatty acylation level on substrate proteins). (B)

Quantification of SIRT2 wildtype and HY mutant mediated decrease in lysine fatty acylation levels on IcsB substrates. The western blot and in-gel fluorescence images are shown in Figure 4B. n=3-4 biological replicates. Data are represented as mean ± SEM. (C – D) The fatty acylation levels of IcsB substrate proteins were higher in SIRT2 knockdown cells. The lysine fatty acylation on Flag-tagged CHMP5, Rac1, and Rac2 in HEK293T (control or SIRT2 KD) cells expressing IcsB (C) or infected with *S. flexneri* (D) was detected by in-gel fluorescence. The quantification of lysine fatty acylation levels (normalized to flag) is show below the images. (E) SIRT6 and SIRT7 cannot remove IcsB mediated lysine fatty acylation. HDAC11 can remove IcsB mediated lysine fatty acylation. In-gel fluorescence detection of lysine fatty acylation of Flag-tagged CHMP5 in HEK293T cells that were co-transfected with Flag-tagged IcsB and SIRT2, empty vector (EV), SIRT6, SIRT7, or HDAC11. (F) Overexpression of SIRT2 removes lysine fatty acylation on CHMP5. In-gel fluorescence detection of lysine fatty acylation on Flag-CHMP5 WT or K7R in HEK293T cells that were also transfected with Flag-IcsB and SIRT2.

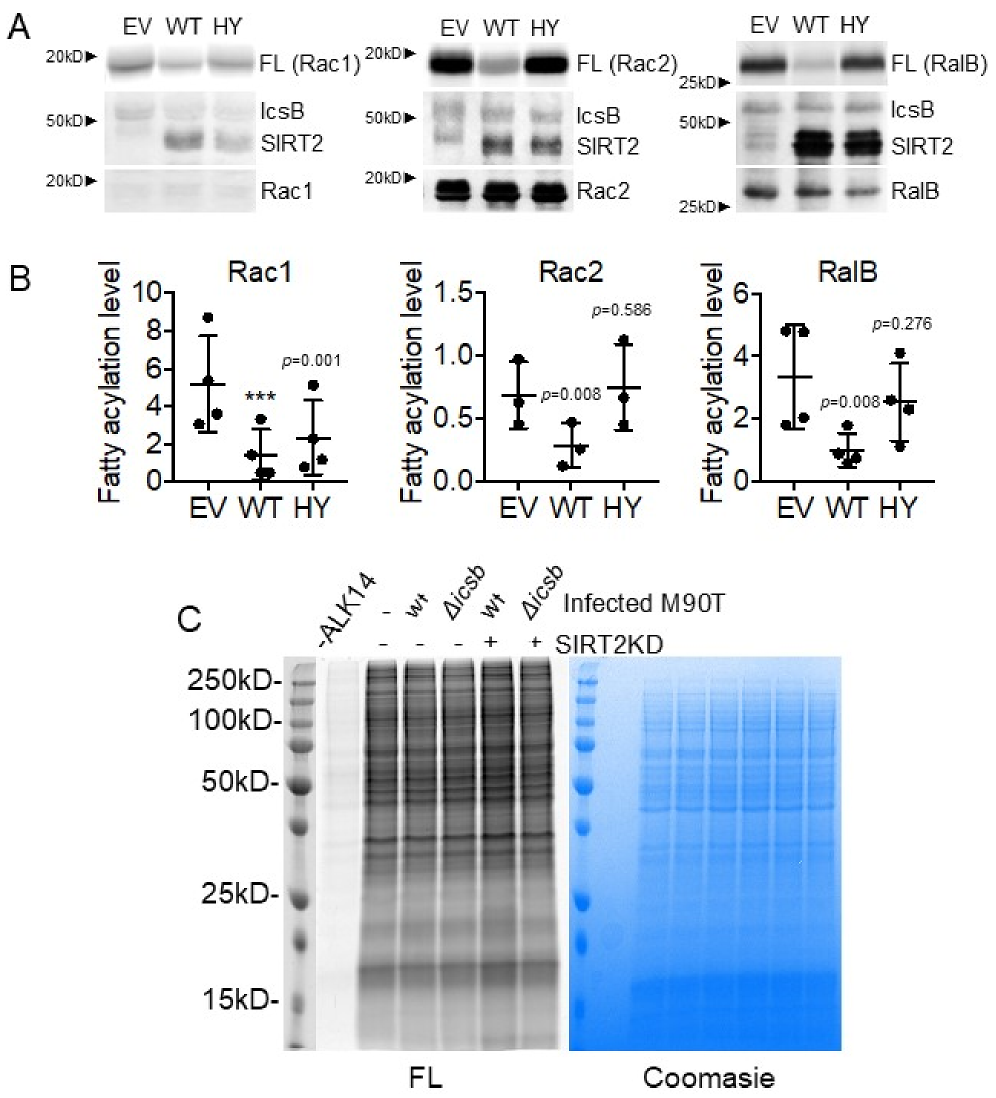

Supplementary Figure 4. The fatty acylation levels of IcsB substrate proteins are higher when IpaJ is deleted.

(A) In-gel fluorescence detection of lysine fatty acylation of Flag-tagged IcsB substrates in HEK293T cells that were also transfected with Flag-tagged IcsB and SIRT2. Representative images from at least 3 independent experiments are shown. EV, empty vector. WT, SIRT2 WT. HY, SIRT2 HY. FL, fluorescence (indicative of lysine fatty acylation level on substrate proteins). Flag, anti-Flag immunoblot (indicative of input level of substrate proteins). (B) Quantification of the relative lysine fatty acylation levels in (A). n=3-4 biological replicates. Data are represented as mean ± SEM. Statistical evaluation was done using unpaired two-tail Student's t test. (C) Fluorescence detection of Global protein fatty acylation in control or SIRT2 KD HEK 293T cells infected with WT or IcsB deletion *S. flexneri* M90T. ****p<0.001.*

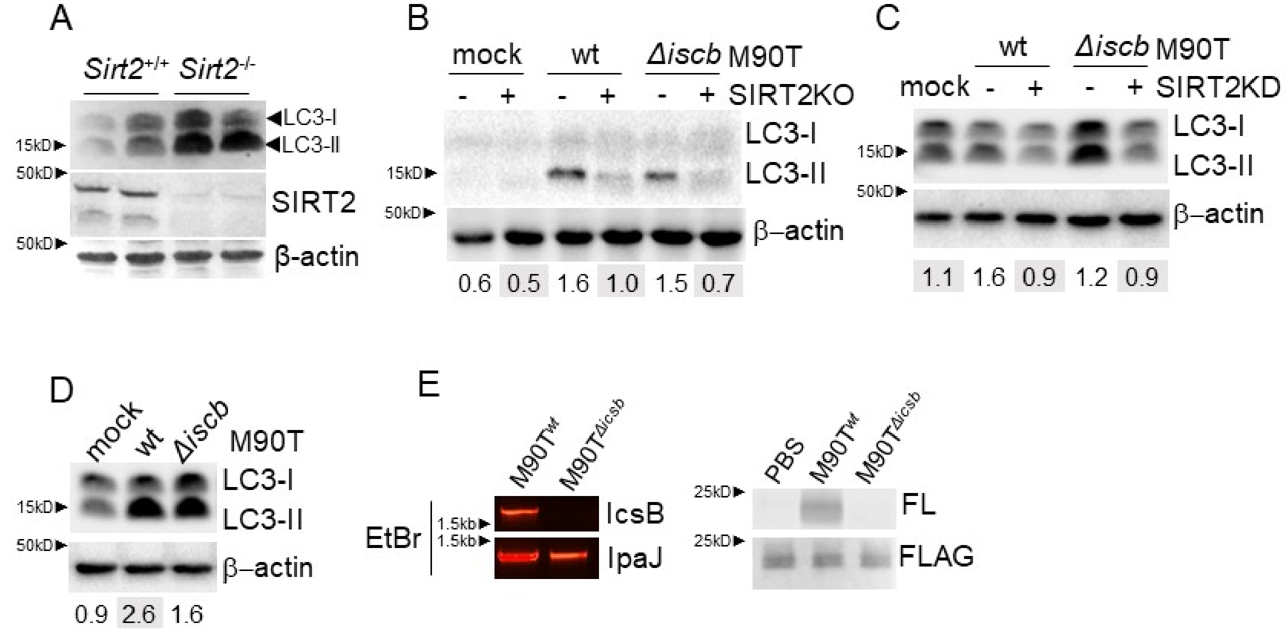

Supplementary Figure 5. SIRT2 suppresses *Shigella* autophagosome escape

(A) Western blots to confirm there is similar basal level autophagy in WT and *Sirt2*$^{-/-}$ MEF cells. (B-C) Autophagy level (measured by ratio of LC3-II to LC3-I) is lower in SIRT2 knockout and knockdown cells upon *S. flexneri* infection. Immunoblotting analysis of LC3 in WT and *Sirt2* knockout MEF cells (B) and control or SIRT2 knockdown A549 cells (C) infected with wildtype or IcsB deletion *S. flexneri* M90T strain at the same MOI. The quantification of LC3-II/LC3-I is shown at the bottom. (D) Autophagy was lower when IpaJ was deleted. Immunoblots of LC3 levels in A549 cells that were infected with mock, or same MOI of M90T wildtype and IpaJ deletion strain are shown. The quantification of LC3-II/LC3-I is shown below the blots. (E) Confirmation of IcsB deletion by PCR using IcsB specific primer (left) and in-gel fluorescence for detecting RhoA lysine fatty acylation (right). FL, fluorescence, RhoA lysine fatty acylation level. Flag, RhoA protein level.

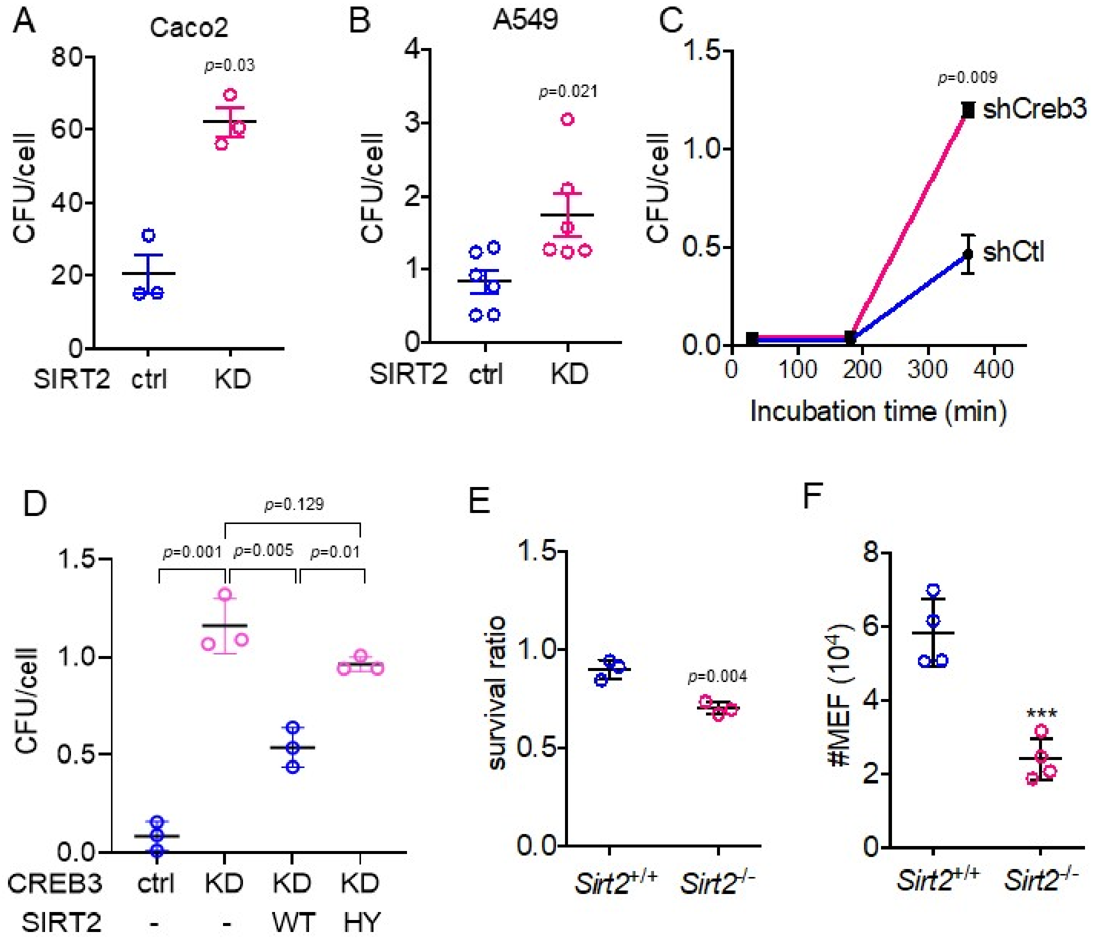

Supplementary Figure 6. SIRT2 restricts *Shigella* infection in cells.
(A – B) SIRT2 restricts intracellular Shigella proliferation in human colon epithelial cells Caco-2 (A) and human lung epithelial cells A549 (B). Control and SIRT2 KD cells were infected with the same number of *S. flexneri* M90T cells for 10 min, then treated with gentamycin to kill extracellular bacteria. Intracellular *S. flexneri* number and mammalian cell number were counted to get CFU/cell. (C) Effect of CREB3 knockout on *S. flexneri* intracellular proliferation. CFU/cell were determined after 10 min of infection and the indicated time of incubation in the presence of 50 µg/ml gentamicin in control and CREB3 knockdown A549 cells. (D) CREB3 KD cells transfected with WT or HY SIRT2 and infected with *S. flexneri* M90T for 10 mins before washing with PBS and replacing the media with 50 µg/ml gentamicin for 6 hours. Cells were collected and CFU/cell determined. (E) MEF cell survival ratio after 5 µg/ml of BFA treatment. (F) Number of MEF cells at the end of the gentamycin killing assay. Statistical evaluation was done using an unpaired two-tailed Student's t test. Data are represented as mean ± SEM with three (A), six (B), three (D), three (E) or four (F) biological replicates. ****$p<0.001$.*

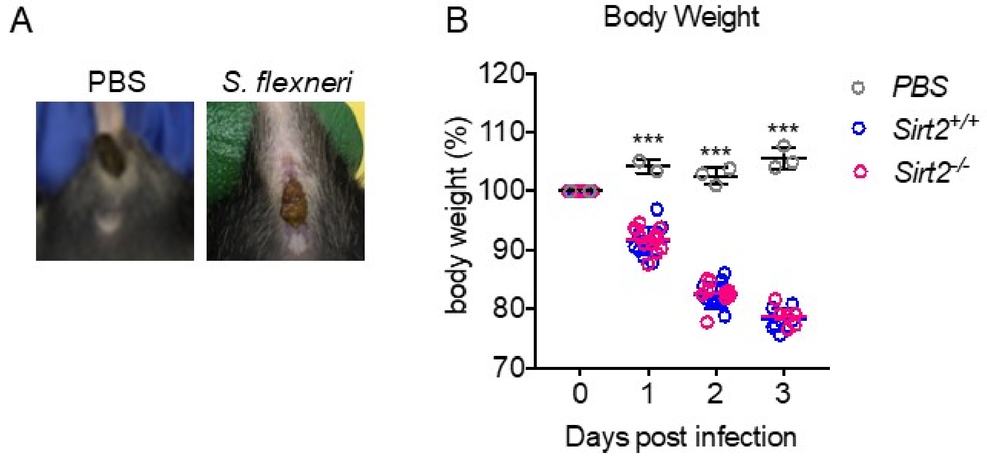

Supplementary Figure 7. *Shigella* infection in an intranasal (i.n.) shigellosis mouse model. (A) Photos of anal region of C57B6/J mice infection with 1 million *S. flexneri* M90T cells for 3 days after intranasal infection, indicating diarrhea. (B) Body weight was monitored after C57B6/J *Sirt2*$^{+/+}$ and *Sirt2*$^{-/-}$ mice were infected with intranasal administration of 1 million wildtype *S. flexneri* M90T cells. n=3, 9, or 12 biological replicates. Statistical evaluation was done using unpaired two-tail Student's t test. Data are represented as mean ± SEM. ***$p<0.001$.

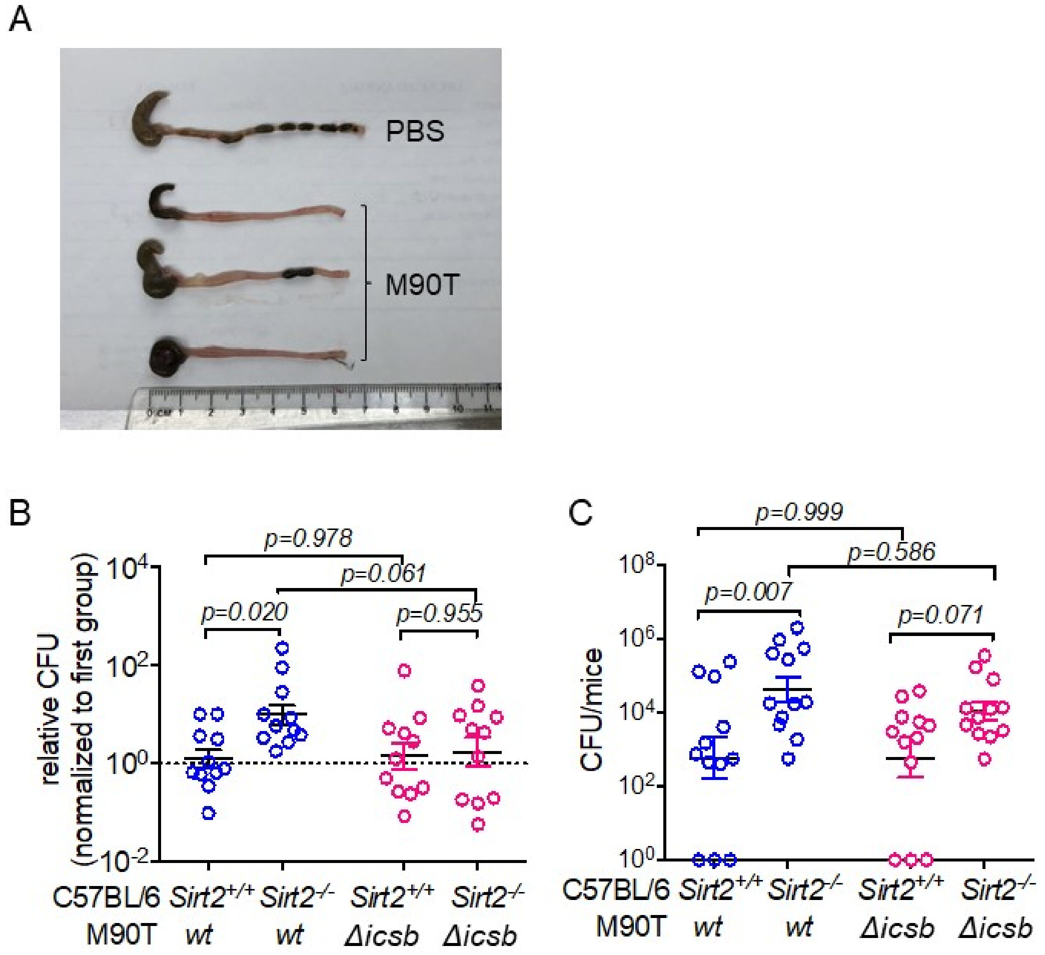

Supplementary Figure 8. SIRT2 restricts *Shigella* infection in an intraperitoneal (i.p.) shigellosis mouse model.

(A) WT C57BL/6J mice colon lengths at 6 hrs post i.p.-infection with *S. flexneri*. (B – C) CFU in liver samples (B) and peritoneal wash samples (C) from *Sirt2*$^{+/+}$ and *Sirt2*$^{-/-}$ C57BL/6J mice infected with 150 million *S. flexneri* M90T wildtype and IcsB deletion cells for 17 hrs. Data are presented as mean ± SEM (n = 11). Statistical evaluation was done using two-way ANOVA.

Table S1. Primers for RT-PCR

Target	Sequence
Human *CREB3*	AAGAGGGGACCCAGATGACT<br>AGGAGGAGGCAGAAGGAGAC
Human *SIRT1*	TAGCCTTGTCAGATAAGGAAGGA<br>ACAGCTTCACAGTCAACTTTGT
Human *SIRT2*	TGCGGAACTTATTCTCCCAGA<br>GAGAGCGAAAGTCGGGGAT
Human *SIRT3*	ACCCAGTGGCATTCCAGAC<br>GGCTTGGGGTTGTGAAAGAAG
Human *SIRT4*	GCTTTGCGTTGACTTTCAGGT<br>CCAATGGAGGCTTTCGAGCA
Human *SIRT5*	GCCATAGCCGAGTGTGAGAC<br>CAACTCCACAAGAGGTACATCG
Human *SIRT6*	CCCACGGAGTCTGGACCAT<br>CTCTGCCAGTTTGTCCCTG
Human *SIRT7*	GACCTGGTAACGGAGCTGC<br>CGACCAAGTATTTGGCGTTCC
Human *HDAC11*	CACGCTCGCCATCAAGTTTC<br>GAAGTCTCGCTCATGCCCATT
Human *ACTB* (internal control)	CATGTACGTTGCTATCCAGGC<br>CTCCTTAATGTCACGCACGAT
Mouse *Creb3*	AAGGCTCCGCTGGACTTAGA<br>TGTGGAAGGGAGTAGTTGTGA
Mouse *Sirt2*	GCCTGGGTTCCCAAAAGGAG<br>GAGCGGAAGTCAGGGATACC
Mouse Actb (internal control)	GGCTGTATTCCCCTCCATCG<br>CCAGTTGGTAACAATGCCATGT
