# Supplementary material for: Golgi stress induces SIRT2 to counteract Shigella infection via defatty-acylation
Source: Nat Commun. 2022 Aug 2;13:4494. doi: 10.1038/s41467-022-32227-x (PMC9345896; doi:10.1038/s41467-022-32227-x)
Supplement: Supplementary file 3 — Reporting Summary [file 41467_2022_32227_MOESM3_ESM.pdf]

Corresponding author(s): *Double-blind peer review submissions: write DBPR and your manuscript number here instead of author names.*

Last updated by author(s): Jun 7, 2022

Reporting Summary

Nature Portfolio wishes to improve the reproducibility of the work that we publish. This form provides structure for consistency and transparency in reporting. For further information on Nature Portfolio policies, see our Editorial Policies and the Editorial Policy Checklist.

Statistics

For all statistical analyses, confirm that the following items are present in the figure legend, table legend, main text, or Methods section.

n/a	Confirmed	
☐	☒	The exact sample size (*n*) for each experimental group/condition, given as a discrete number and unit of measurement
☐	☒	A statement on whether measurements were taken from distinct samples or whether the same sample was measured repeatedly
☐	☒	The statistical test(s) used AND whether they are one- or two-sided<br>*Only common tests should be described solely by name; describe more complex techniques in the Methods section.*
☒	☐	A description of all covariates tested
☒	☐	A description of any assumptions or corrections, such as tests of normality and adjustment for multiple comparisons
☐	☒	A full description of the statistical parameters including central tendency (e.g. means) or other basic estimates (e.g. regression coefficient) AND variation (e.g. standard deviation) or associated estimates of uncertainty (e.g. confidence intervals)
☐	☒	For null hypothesis testing, the test statistic (e.g. *F*, *t*, *r*) with confidence intervals, effect sizes, degrees of freedom and *P* value noted<br>*Give P values as exact values whenever suitable.*
☒	☐	For Bayesian analysis, information on the choice of priors and Markov chain Monte Carlo settings
☒	☐	For hierarchical and complex designs, identification of the appropriate level for tests and full reporting of outcomes
☒	☐	Estimates of effect sizes (e.g. Cohen's *d*, Pearson's *r*), indicating how they were calculated

Our web collection on statistics for biologists contains articles on many of the points above.

Software and code

Policy information about availability of computer code

Data collection	Gel and immunoblot images were collected with Biorad Chemidoc MP. qPCR data was collected with Applied Biosystems Quantstudio 7 Flex.
Data analysis	Data analysis was done with GraphPad Prism v9 and FIJI (ImageJ) v1.53q.

For manuscripts utilizing custom algorithms or software that are central to the research but not yet described in published literature, software must be made available to editors and reviewers. We strongly encourage code deposition in a community repository (e.g. GitHub). See the Nature Portfolio guidelines for submitting code & software for further information.

Data

Policy information about availability of data

All manuscripts must include a data availability statement. This statement should provide the following information, where applicable:

- Accession codes, unique identifiers, or web links for publicly available datasets
- A description of any restrictions on data availability
- For clinical datasets or third party data, please ensure that the statement adheres to our policy

Unless otherwise stated, data are collected as experimental replicates. Uncropped blots and raw values used to create graphs are available in the Source Data file. Additionally, datasets generated during and/or analyzed during the current study are available from the corresponding author on reasonable request.

Human research participants

Policy information about studies involving human research participants and Sex and Gender in Research.

Reporting on sex and gender	*Use the terms sex (biological attribute) and gender (shaped by social and cultural circumstances) carefully in order to avoid confusing both terms. Indicate if findings apply to only one sex or gender; describe whether sex and gender were considered in study design whether sex and/or gender was determined based on self-reporting or assigned and methods used. Provide in the source data disaggregated sex and gender data where this information has been collected, and consent has been obtained for sharing of individual-level data; provide overall numbers in this Reporting Summary. Please state if this information has not been collected. Report sex- and gender-based analyses where performed, justify reasons for lack of sex- and gender-based analysis.*
Population characteristics	*Describe the covariate-relevant population characteristics of the human research participants (e.g. age, genotypic information, past and current diagnosis and treatment categories). If you filled out the behavioural & social sciences study design questions and have nothing to add here, write "See above."*
Recruitment	*Describe how participants were recruited. Outline any potential self-selection bias or other biases that may be present and how these are likely to impact results.*
Ethics oversight	*Identify the organization(s) that approved the study protocol.*

Note that full information on the approval of the study protocol must also be provided in the manuscript.

Field-specific reporting

Please select the one below that is the best fit for your research. If you are not sure, read the appropriate sections before making your selection.

☒ Life sciences ☐ Behavioural & social sciences ☐ Ecological, evolutionary & environmental sciences

For a reference copy of the document with all sections, see nature.com/documents/nr-reporting-summary-flat.pdf

Life sciences study design

All studies must disclose on these points even when the disclosure is negative.

Sample size	No calculations were done to determine sample size. Sample sizes were chosen to observe average effect size and eliminate experimental variability. Some experiments have higher intrinsic variability than others and more samples were used. For example, cell culture experiments have low variability and so three experimental replicates is sufficient to observe and average effect. Mouse experiments have high variability so more replicates were used. The sample sizes are sufficient as they demonstrate statistically significant differences in line with experimental observations.
Data exclusions	No data was excluded.
Replication	Some key experimental findings (Figures 3C, 7D, and 8C) were replicated one or two times independently by other lab members. These replications were successful and are included in the manuscript. For other experiments, replicates were generated create by one of the authors.
Randomization	The identity of samples was known to experimenters, but samples were processed systematically and concurrently for each experimental replication to prevent experimental error or bias. Generation of additional experimental replicates with similar results and additional experiments based on these results confirmed findings. Additionally, datasets included internal controls.
Blinding	Blinding was not necessary as the nature of the experimental results were objective. Inclusion of source files with this manuscript helps to verify this.

Reporting for specific materials, systems and methods

We require information from authors about some types of materials, experimental systems and methods used in many studies. Here, indicate whether each material, system or method listed is relevant to your study. If you are not sure if a list item applies to your research, read the appropriate section before selecting a response.

Materials & experimental systems

n/a	Involved in the study
☐	☒ Antibodies
☐	☒ Eukaryotic cell lines
☒	☐ Palaeontology and archaeology
☐	☒ Animals and other organisms
☒	☐ Clinical data
☒	☐ Dual use research of concern

Methods

n/a	Involved in the study
☒	☐ ChIP-seq
☒	☐ Flow cytometry
☒	☐ MRI-based neuroimaging

Antibodies

Antibodies used	The following antibodies were purchased from Cell Signaling Technology (Danvers, MA): SIRT2 (D4050, #12650), PARP (#9542S), LC3 (#3868S), and anti-rabbit IgG HRP-linked Antibody (#7074S). The following antibodies were from Santa Cruz: β-actin (C-4, sc-47778), GRP78 (H-129, sc-13968). Anti-Flag M2 antibody conjugated with horseradish peroxidase (A8592) and anti-flag M2 affinity gel (A220) were purchased from Sigma. Anti-CREB3 (ab180119) was from Abcam (Cambridge, MA). Anti-CREB3 antibody for ChIP was purchased from Proteintech (11275-1-AP). Cy3-conjugated goat anti-rabbit IgG (H + L) was purchased from ThermoFisher Scientific (A10520).
Validation	SIRT2 was validated by blotting lysates from control and SIRT2 knockdown cells. In mouse and human samples, bands of expected molecular weight decreased in intensity with SIRT2 knockdown. PARP was validated by blotting lysates from cells treated with and without etoposide to see protein cleavage (https://www.cellsignal.com/products/primary-antibodies/parp-antibody/9542). LC3 was validated by blotting lysates cells treated with and without chloroquine to see increased LC3-II (https://www.cellsignal.com/products/primary-antibodies/lc3b-d11-xp-rabbit-mab/3868). β-actin was validated lysates from serval different cell lines (https://www.scbt.com/p/beta-actin-antibody-c4). GRP78 was validated by blotting lysates of cells treated with compounds that increase GRP78 (Fig 1C). A band of expected molecular weight increased in intensity. Flag M2 was validated by blotting lysates from cells transfected with and without flag-tagged constructs. CREB3 (Abcam) was validated by blotting lysates from control and CREB3 knockdown cells (Fig 2C). A band of expected molecular weight decreased in intensity with CREB3 knockdown. CREB3 (Proteintech) was validated by blotting lysates from control and CREB3 knockdown cells. A band of expected molecular weight decreased in intensity with CREB3 knockdown.

Eukaryotic cell lines

Policy information about cell lines and Sex and Gender in Research

Cell line source(s)	MCF7: female breast mammary cells. HeLa: female ovarian cells. MDA-MB-231: female breast ovarian cells. A2780: female ovarian cells. HeyA8: female ovarian cells. MEF: mouse embryonic fibroblast both male and female. A549: male epithelial cells. HEK293T: human embryonic kidney cells both male and female. BMDM: mouse bone marrow derived macrophages both male and female.
Authentication	Cell lines were purchased authenticated from suppliers via STR profiling (ATCC). Independent authentication was not done. BMDMs were isolated in house via well-established techniques and were not otherwise validated.
Mycoplasma contamination	Cells were not tested for mycoplasma contamination.
Commonly misidentified lines (See ICLAC register)	No commonly misidentified cell lines were used.

Animals and other research organisms

Policy information about studies involving animals; ARRIVE guidelines recommended for reporting animal research, and Sex and Gender in Research

Laboratory animals	C57/B6J mice both wild-type and SIRT2 knockout between the ages of 6-8 weeks were used for this study. Mice were housed in a facility with a 14-light, 10-hour dark cycle. The facility maintained a temperature of approximately 22 degrees Celsius an approximately 50% humidity.
Wild animals	The study did not involve wild animals.
Reporting on sex	Care was taken even disperse sexes between experimental groups. Findings were consistent between sexes so no sex-based analysis was done.
Field-collected samples	The study did not involve field-collected samples.
Ethics oversight	All animal experiments were approved by Cornell University's Institutional Animal Care and Use Committee.

Note that full information on the approval of the study protocol must also be provided in the manuscript.
