# Supplementary material for: Golgi stress induces SIRT2 to counteract Shigella infection via defatty-acylation
Source: Nat Commun. 2022 Aug 2;13:4494. doi: 10.1038/s41467-022-32227-x (PMC9345896; doi:10.1038/s41467-022-32227-x)

Fig. 1a

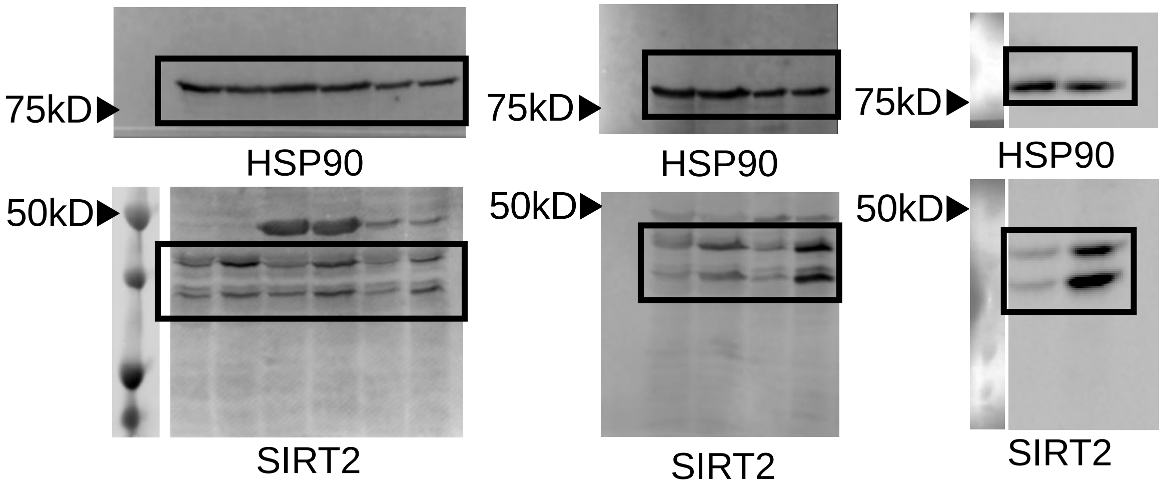

Fig. 1e

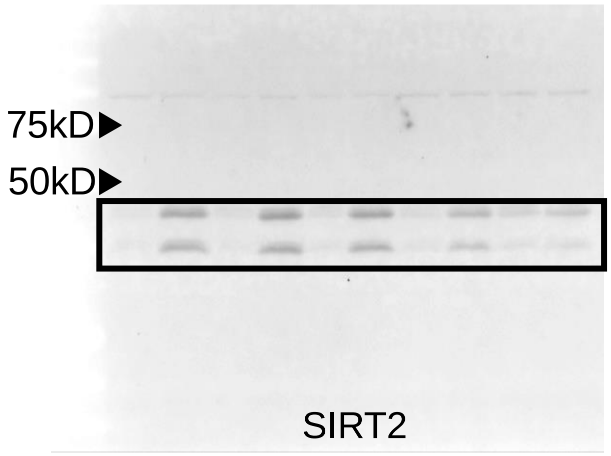

Fig. 1c

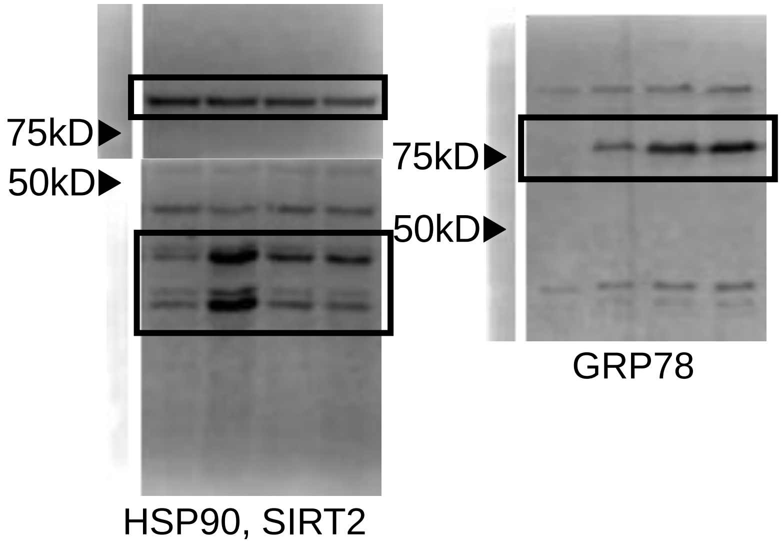

Fig. 1f

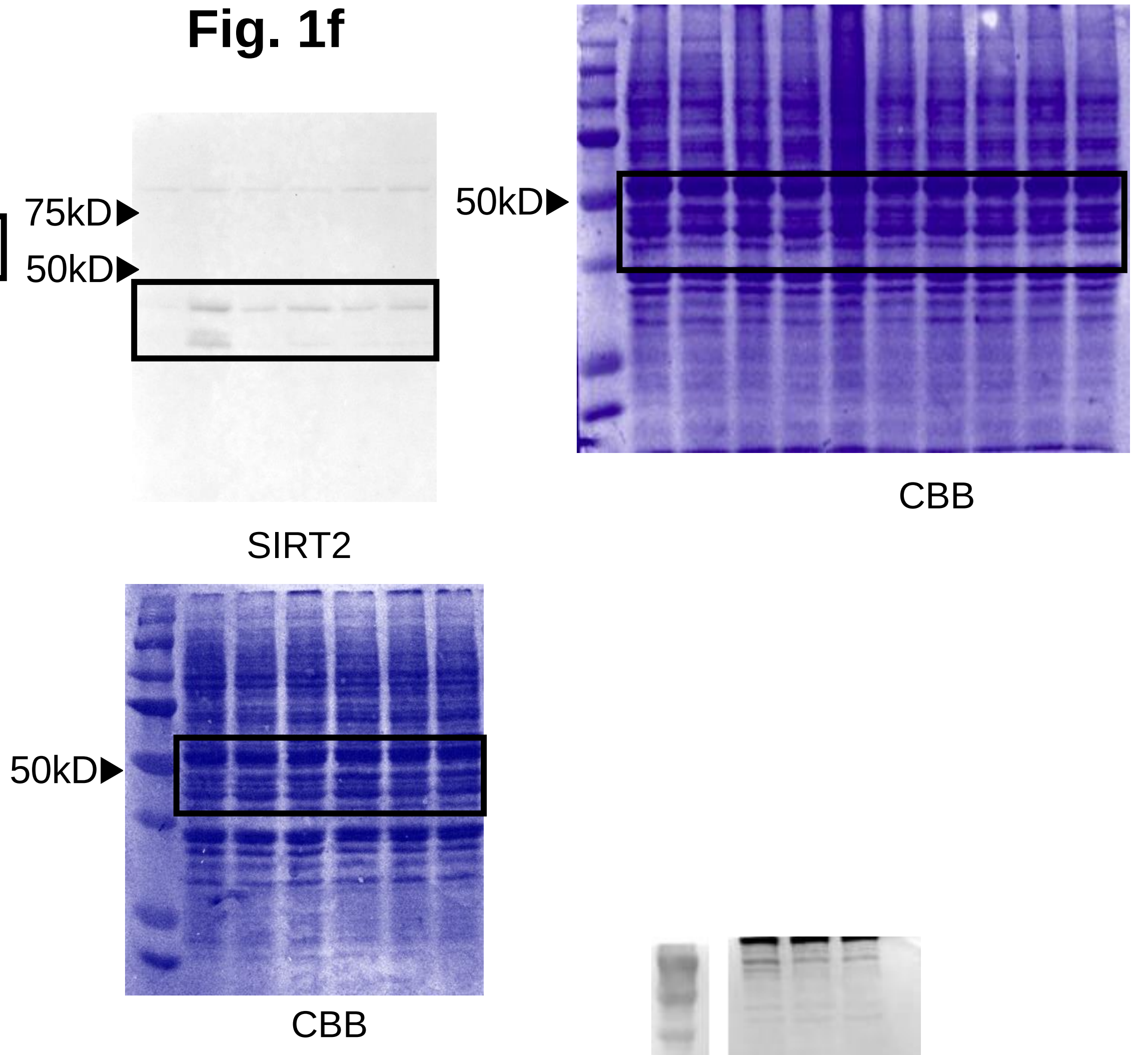

Fig. 1h

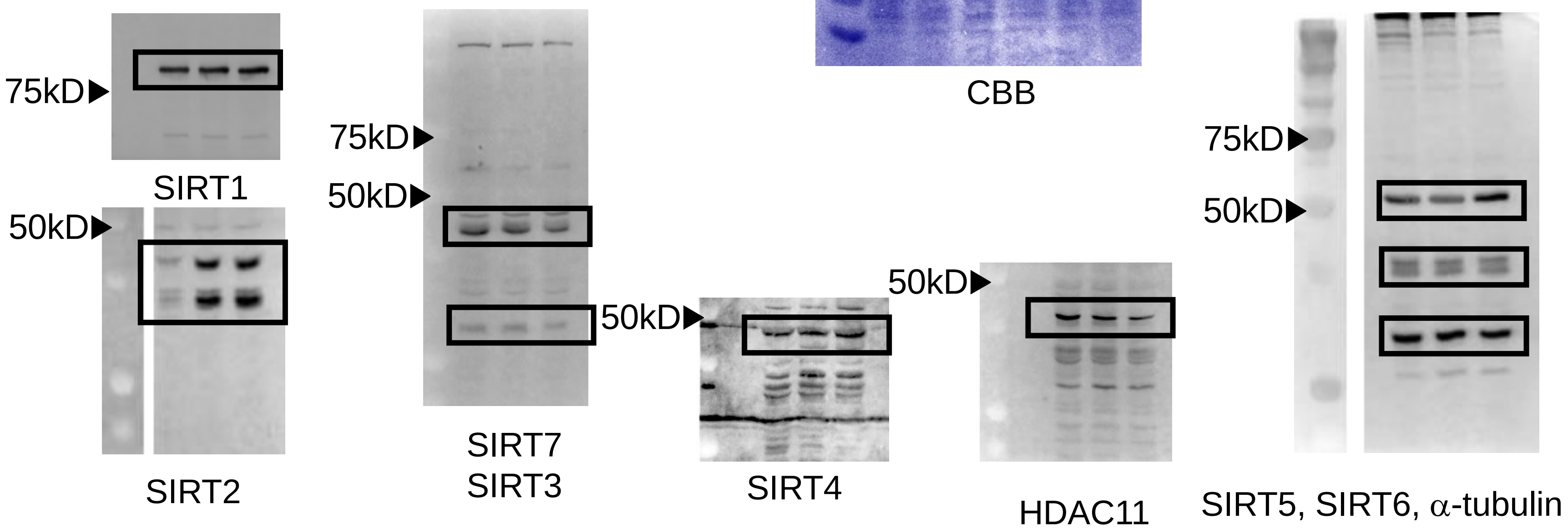

Extended Fig. 2a

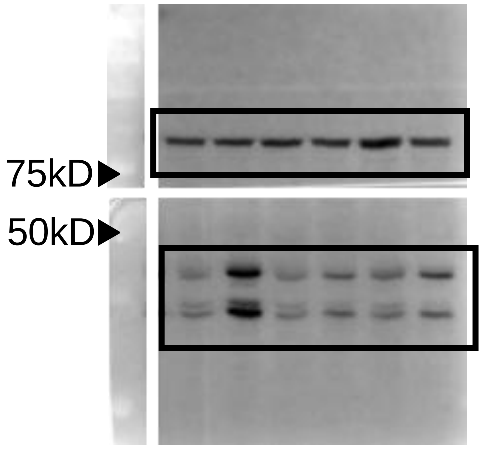

HSP90, SIRT2

Fig. 2c

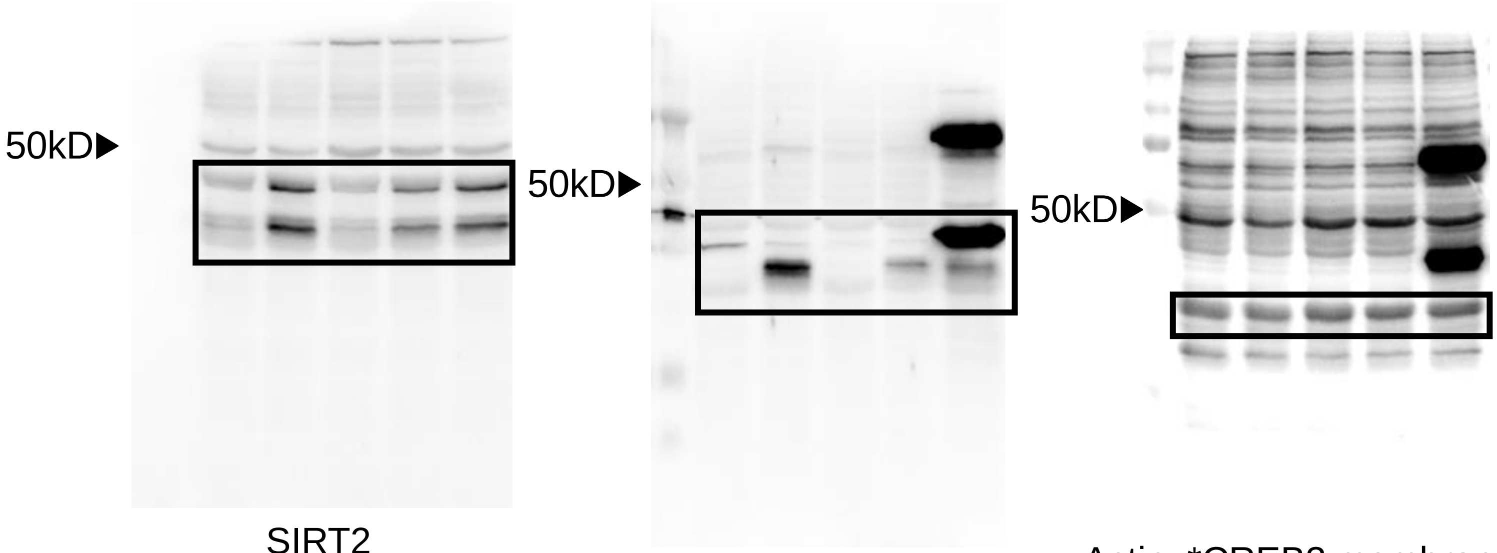

SIRT2

CREB3

Actin *CREB3 membrane reblot

Fig. 2e

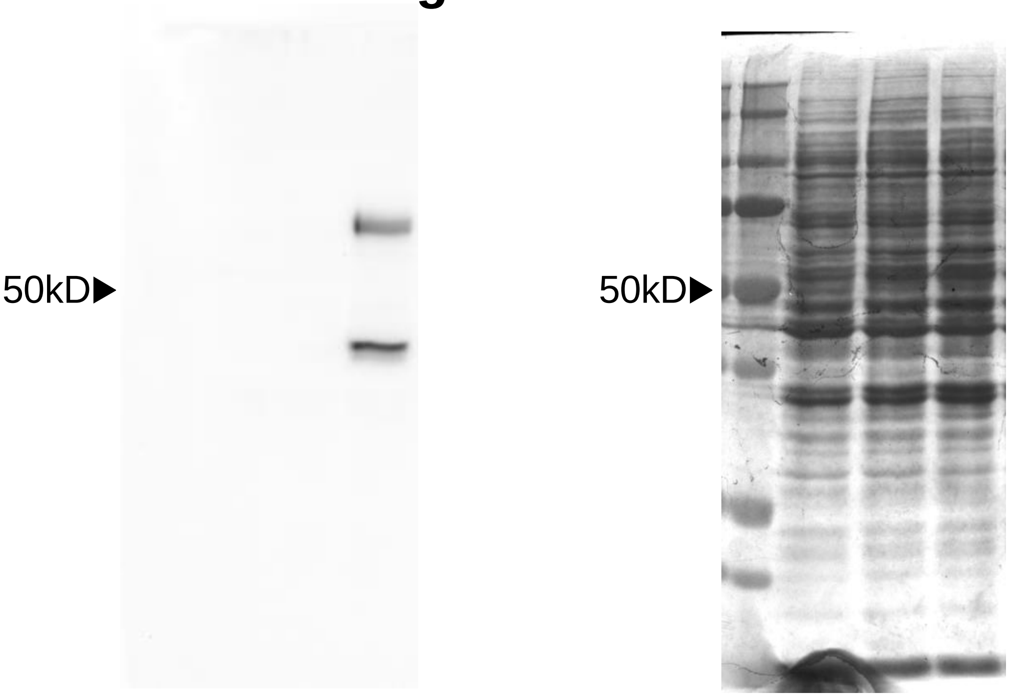

Flag

Coomasie

Fig. 3a

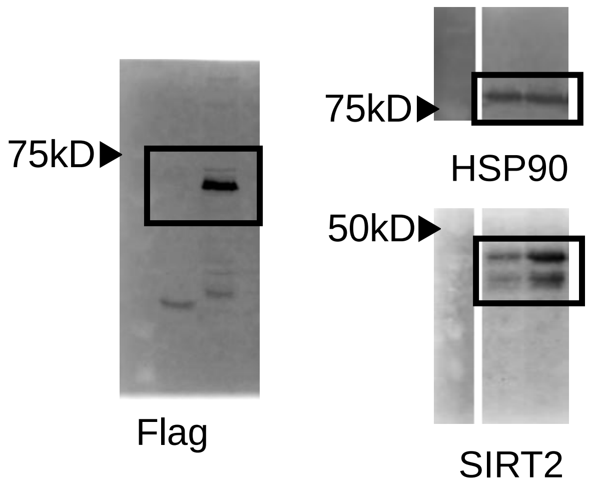

Fig. 3b

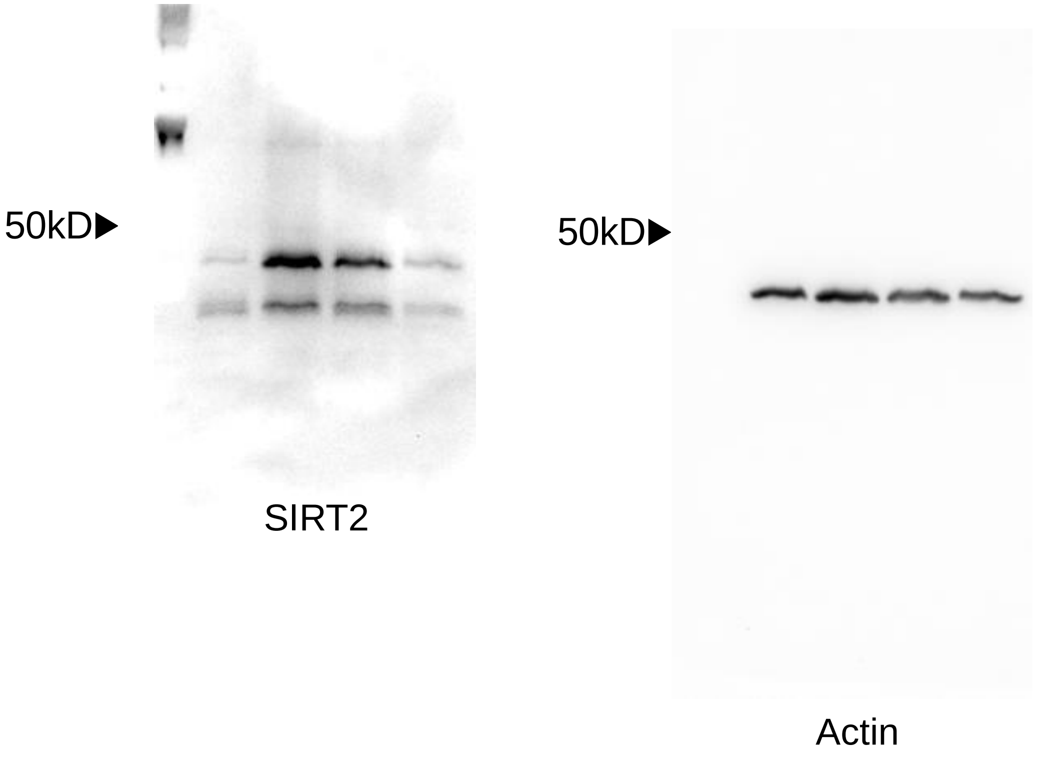

Fig. 4a

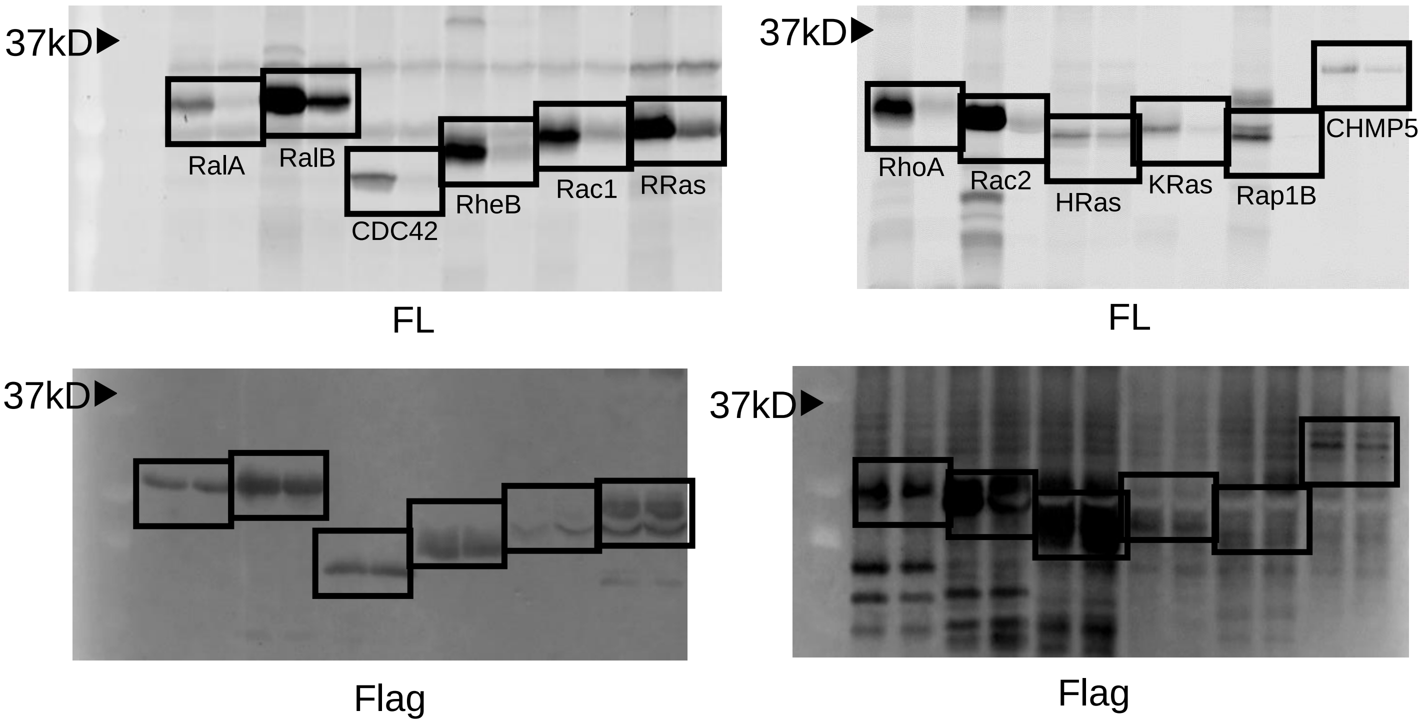

Fig. 4b

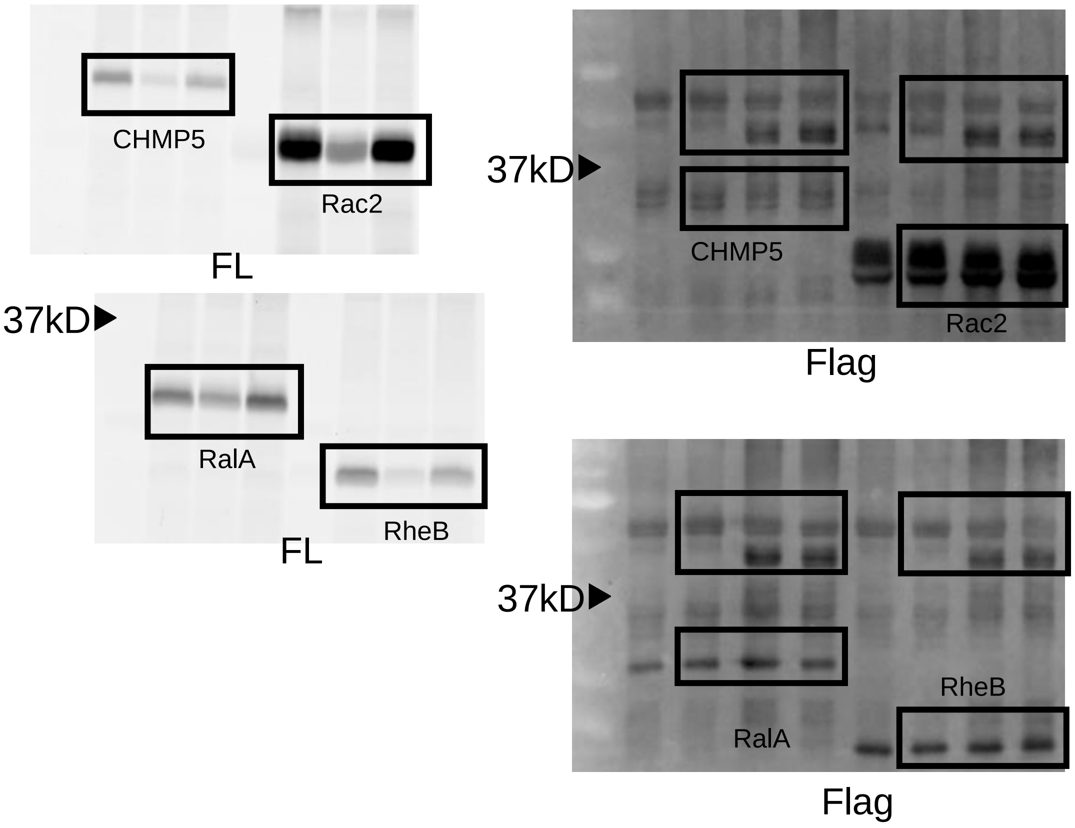

Fig. 5d

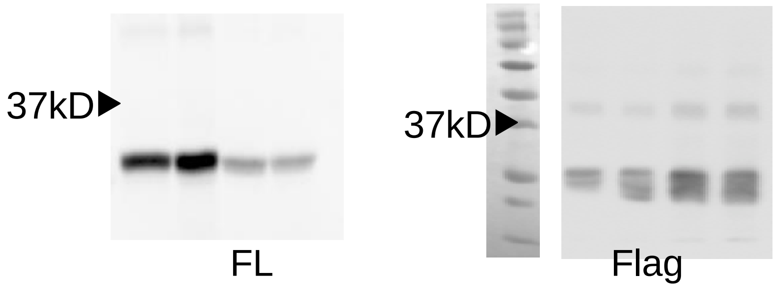

Fig. 5a

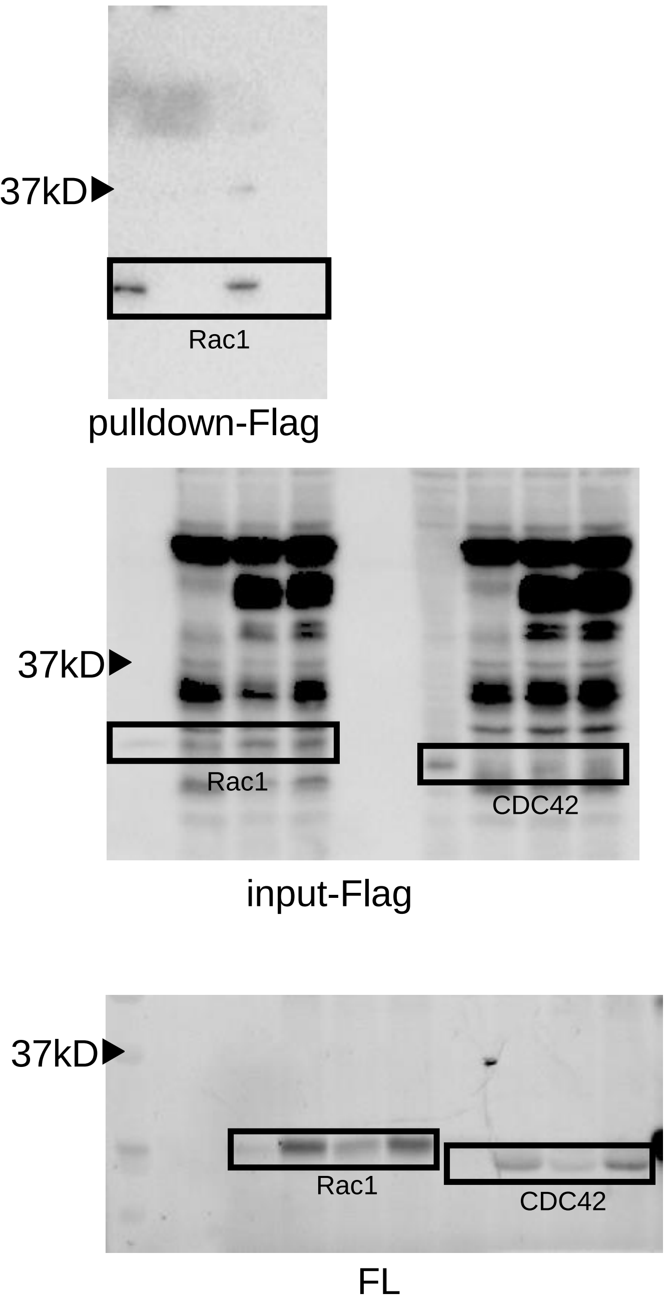

Fig. 5b

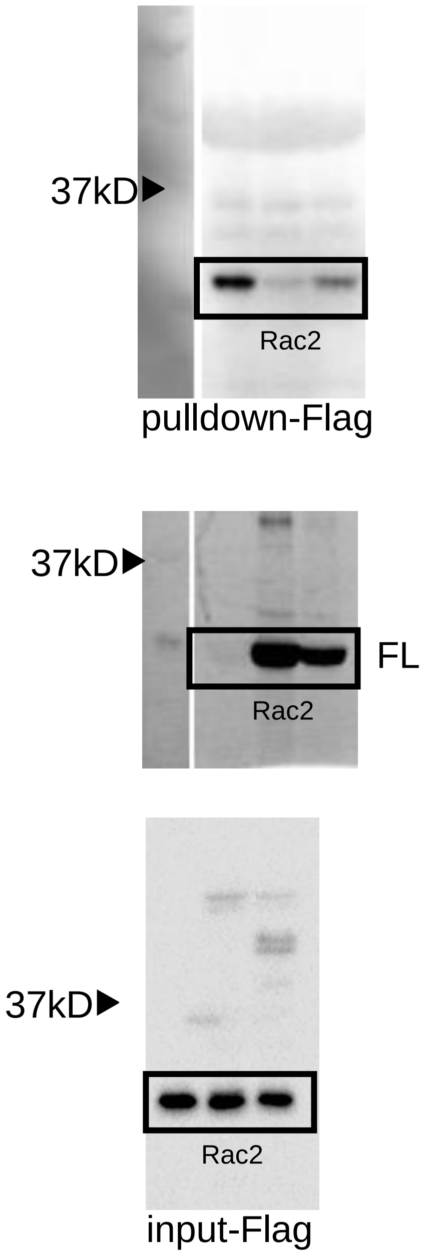

Fig. 5c

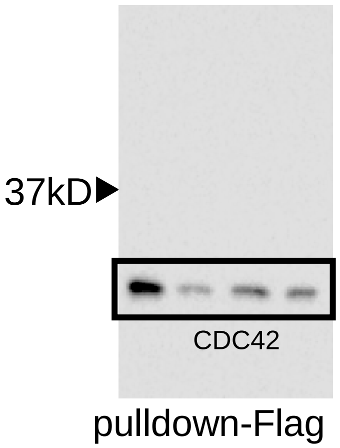

Extended Fig. 2a

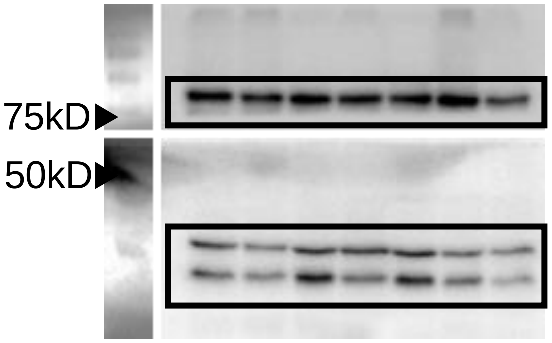

HSP90, SIRT2

Extended Fig. 2b

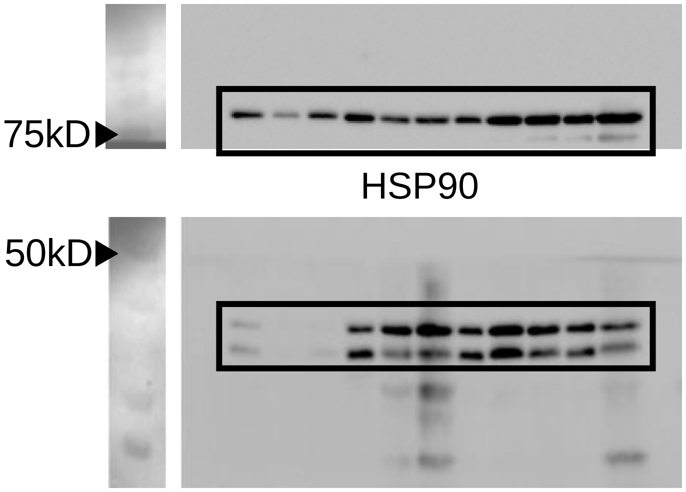

SIRT2

Extended Fig. 2e

Extended Fig. 2c

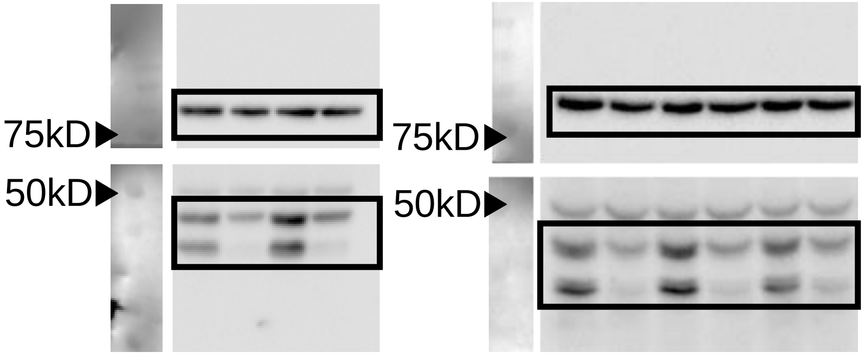

HSP90, SIRT2 HSP90, SIRT2

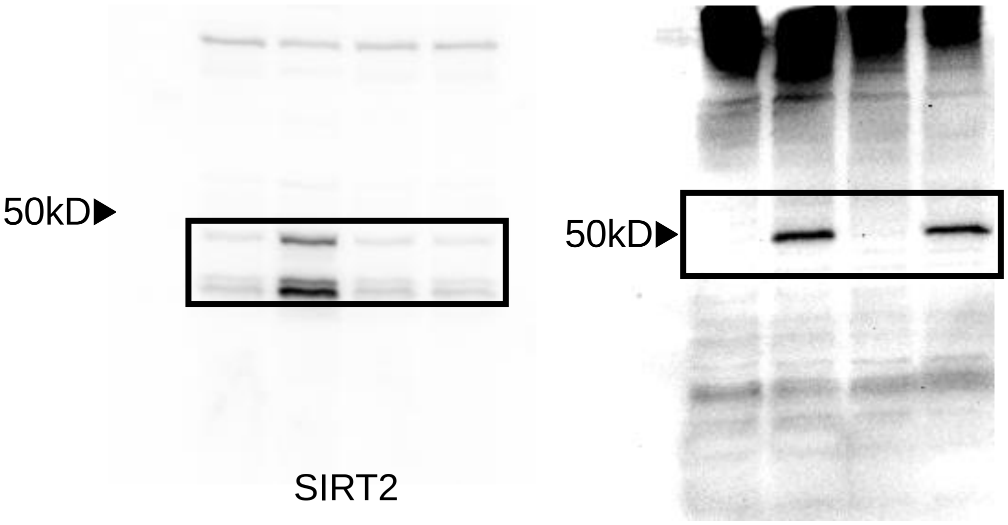

SIRT2

FLAG

Extended Fig. 2f

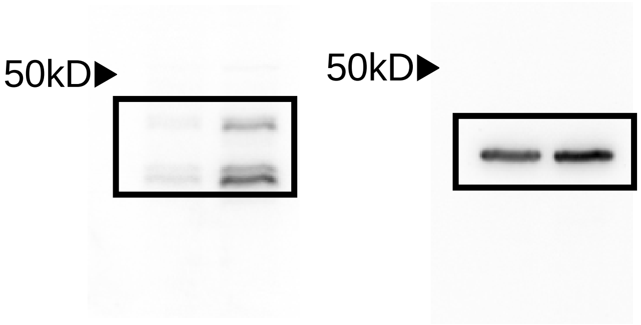

SIRT2 Actin

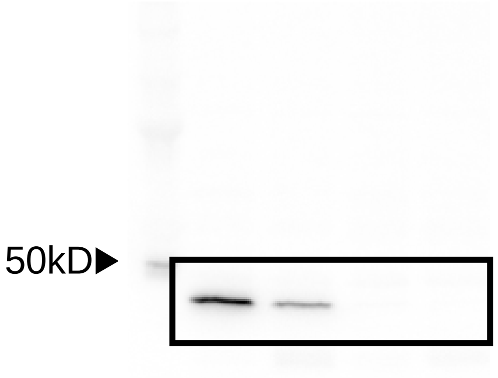

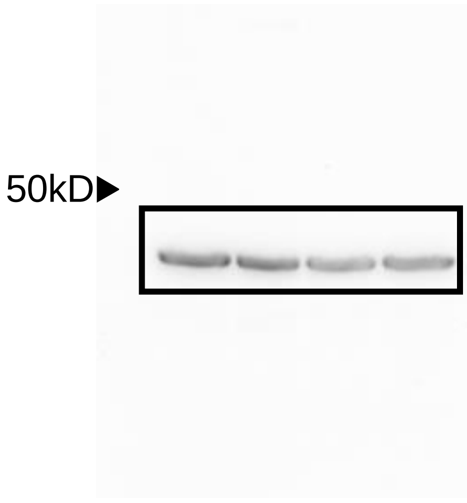

Extended Fig. 2g

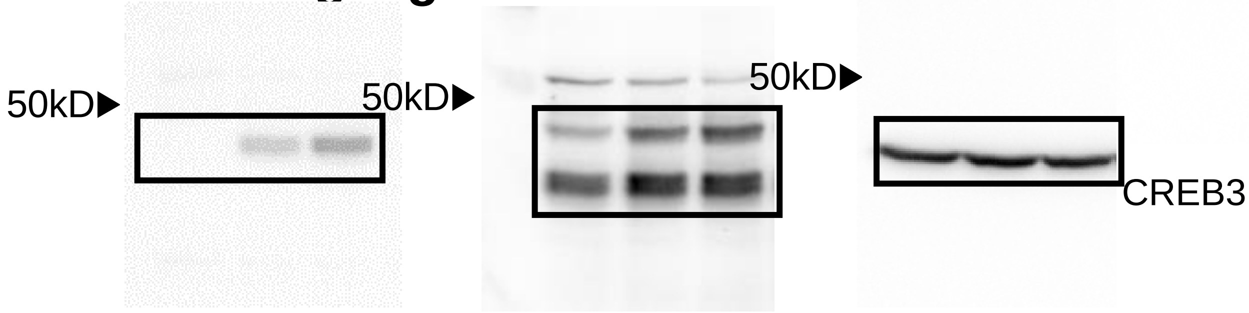

CREB3

Actin

Flag SIRT2 Actin

Extended Fig. 3a

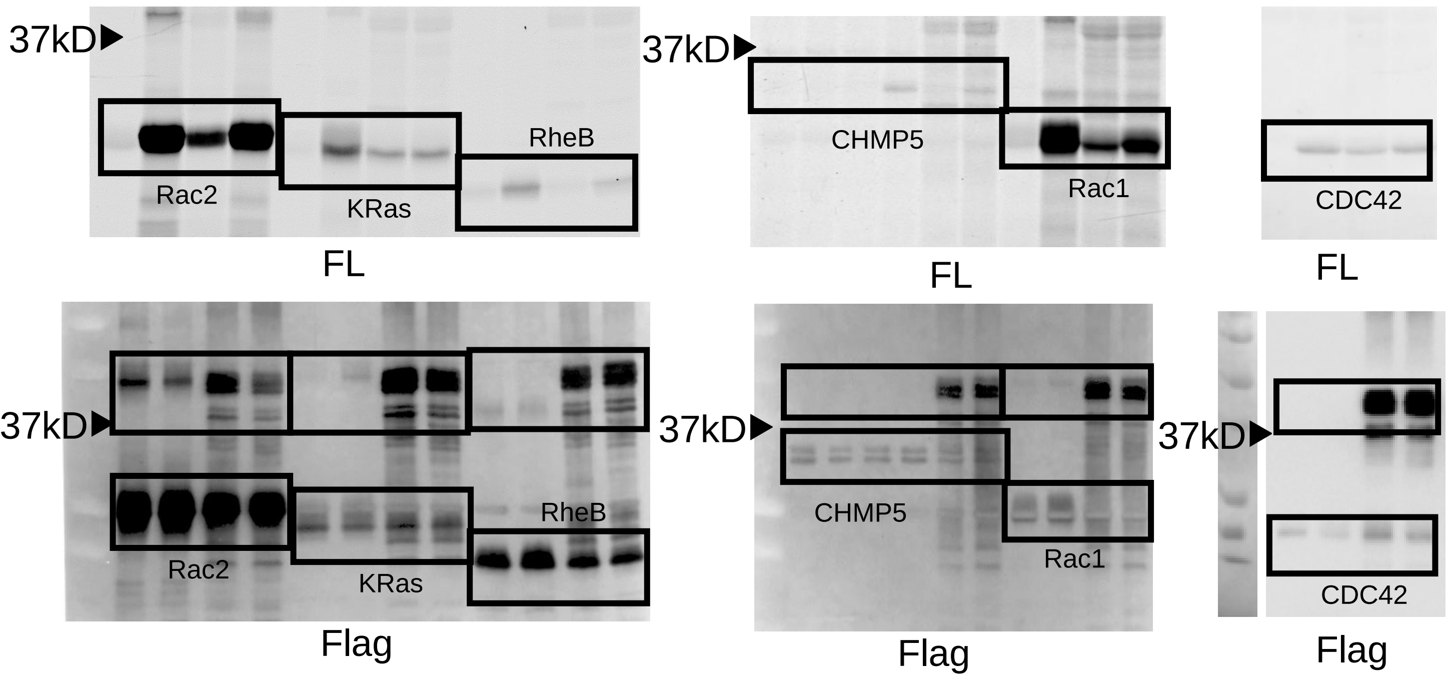

Extended Fig. 3c

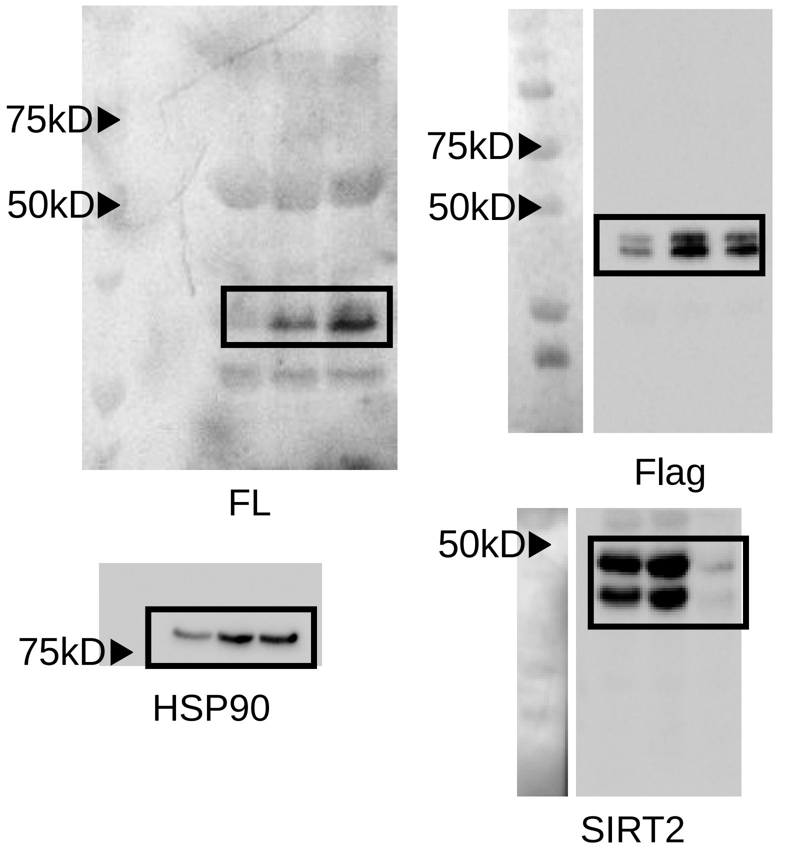

Extended Fig. 3d

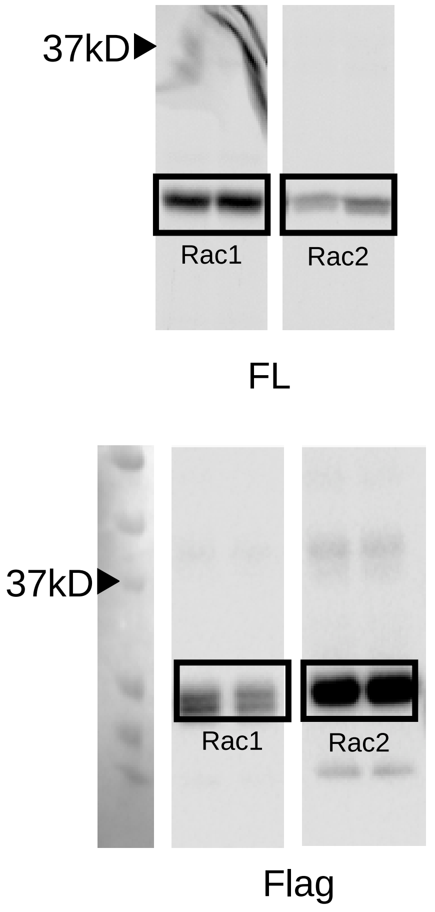

Extended Fig. 3e

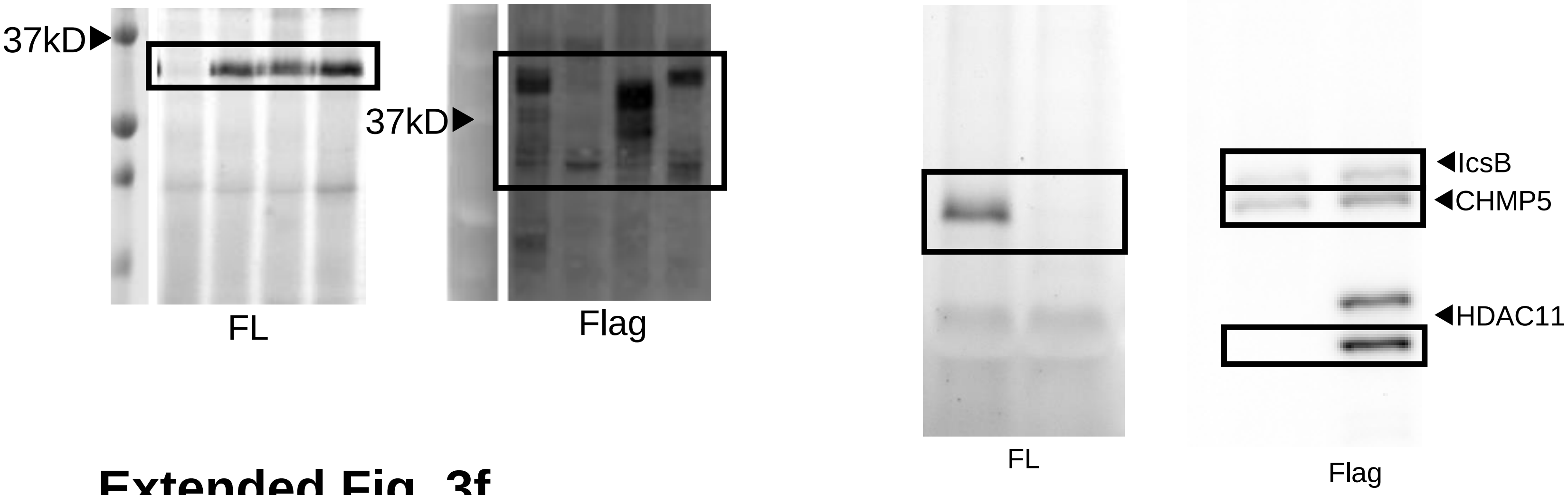

Extended Fig. 3f

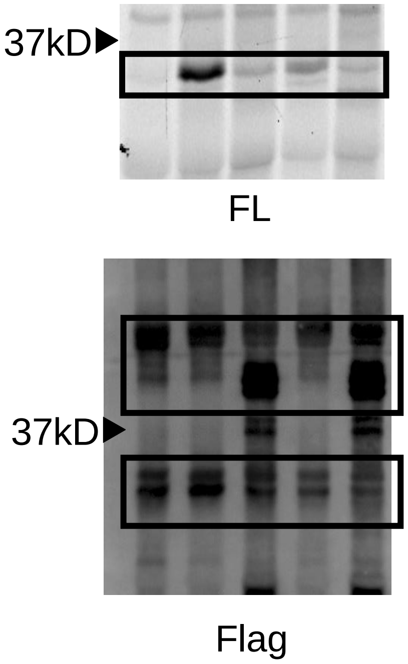

Extended Fig. 4a

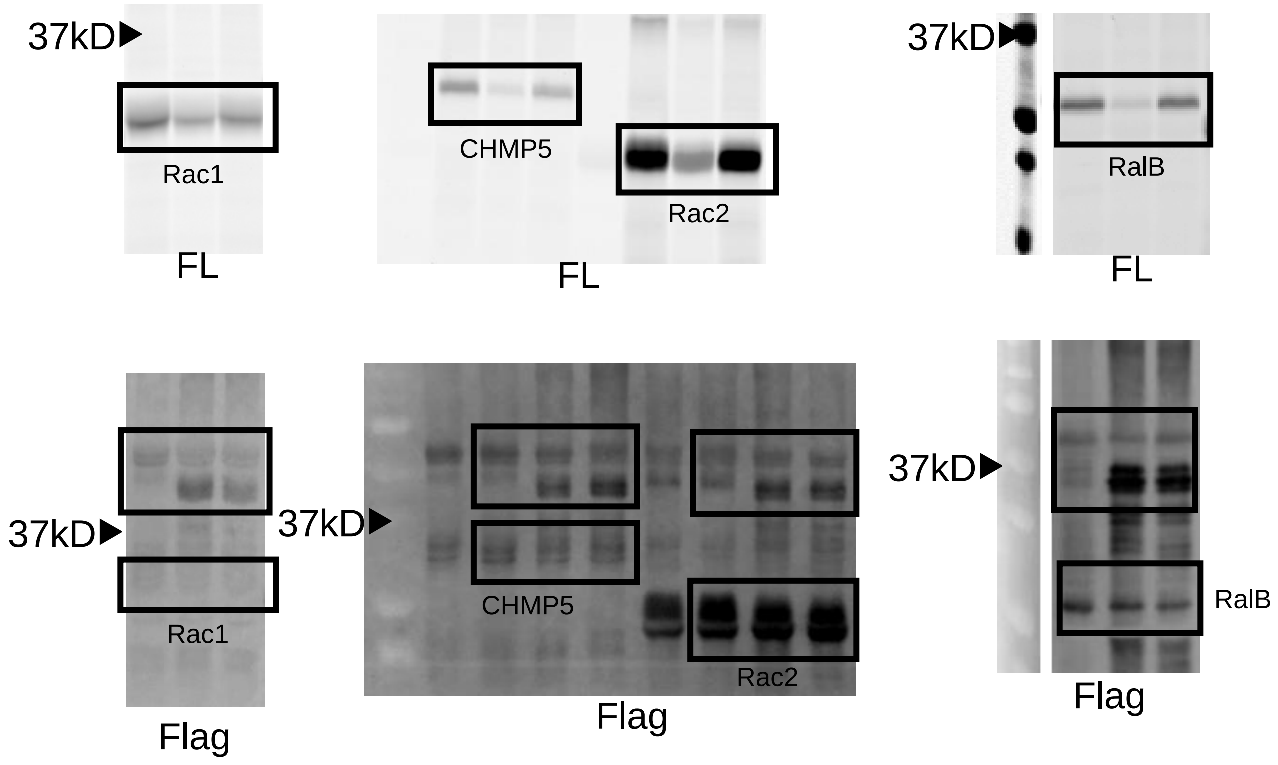

Extended Fig. 4b

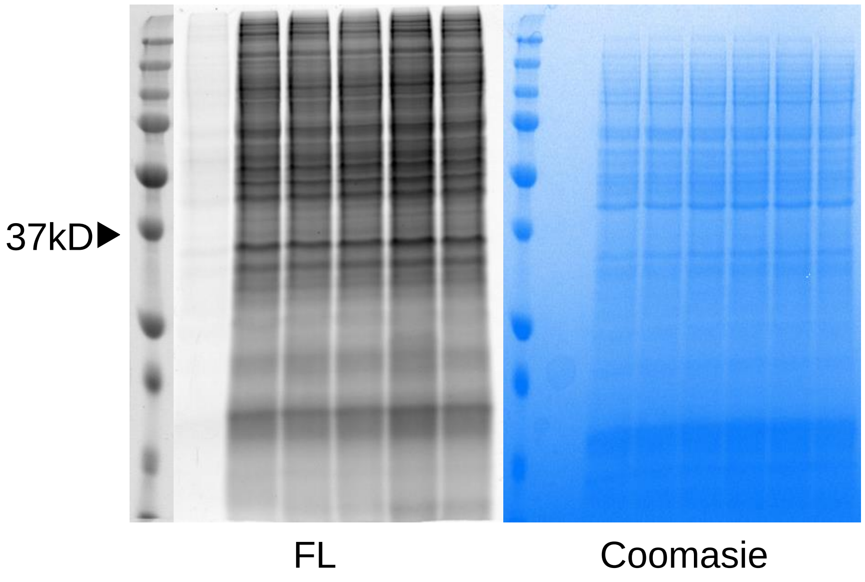

Extended Fig. 5a

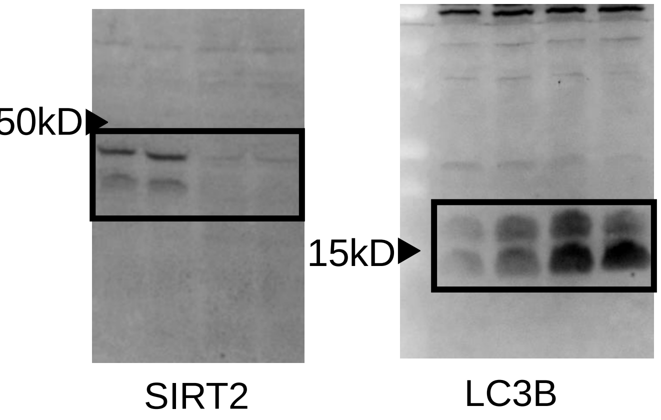

Extended Fig. 5b

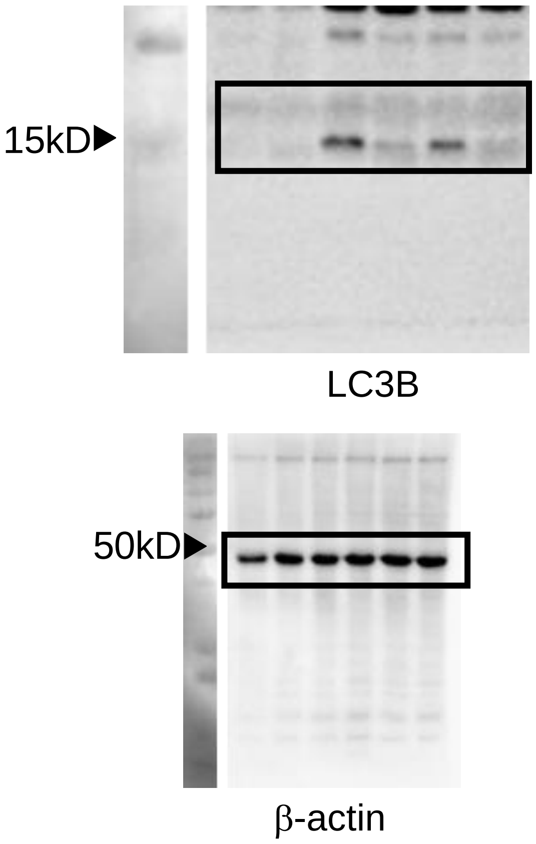

Extended Fig. 5c

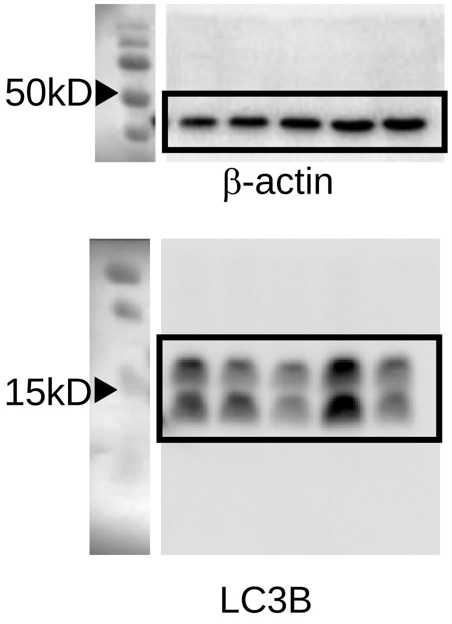

Extended Fig. 5d

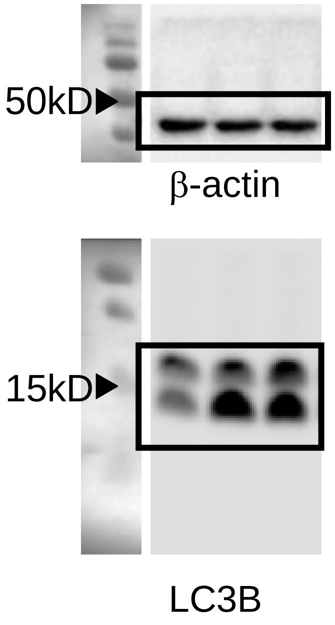

Extended Fig. 5e

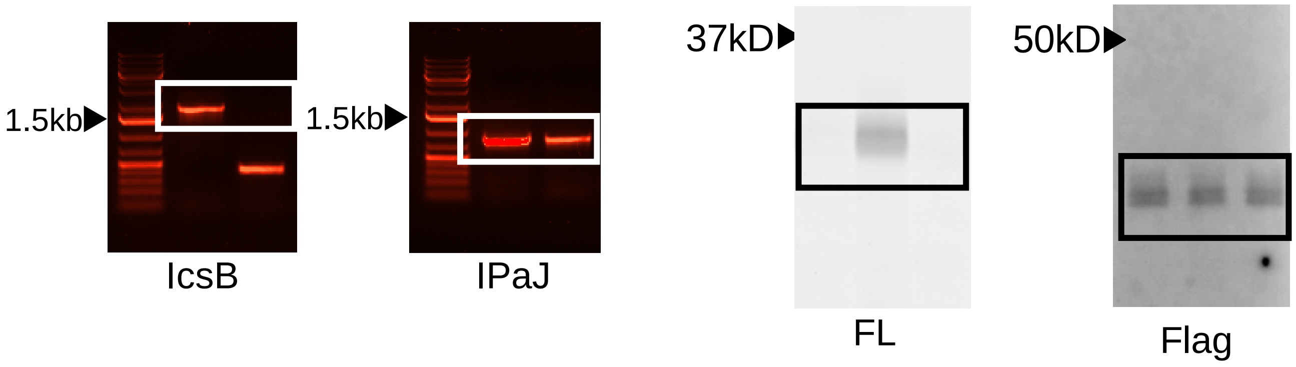

Supplement: Supplementary file 4 — Source Data [file 41467_2022_32227_MOESM4_ESM.zip › Lin_Source Data/Source data_blots and gels.pdf]
